# Masquelet Technique and Arthrodesis for Digit Salvage of the Finger in Traumatic Bone Loss and Osteomyelitis: A Case Report

**DOI:** 10.7759/cureus.26773

**Published:** 2022-07-12

**Authors:** Parker L Brush, Gregory R Toci, Nicholas C Semenza, Daniel Fletcher, Pedro Beredjiklian, Daren Aita

**Affiliations:** 1 Division of Hand Surgery, Rothman Orthopaedic Institute, Philadelphia, USA; 2 Department of Orthopaedic Surgery, Drexel University College of Medicine, Philadelphia, USA

**Keywords:** finger reconstruction, ballistic injuries, bone loss, distal interphalangeal joint arthrodesis, osteo-myelitis, induced-membrane technique, masquelet technique

## Abstract

Use of the Masquelet technique in the hand is uncommon, particularly for cases complicated by osteomyelitis. This case report describes a patient who was advised to proceed with digital amputation following the traumatic segmental bone loss with a non-salvageable distal interphalangeal joint surface complicated by osteomyelitis but refused amputation and requested alternative treatment. We suggested and performed the Masquelet procedure and arthrodesis to salvage the digit. The first stage consisted of surgical debridement and placement of an antibiotic cement spacer, and the second stage included the replacement of the antibiotic cement spacer with an iliac crest autograft and arthrodesis eight weeks after the primary procedure. The Masquelet technique led to the resolution of osteomyelitis, successful osseous union, finger ray salvage, and distal interphalangeal joint arthrodesis.

## Introduction

The Masquelet technique, also known as the induced membrane technique, is a staged method of autologous bone grafting used to treat segmental bone loss from trauma as well as septic and aseptic nonunion in the upper and lower extremities [1]. In 2019, Masquelet et al. reported cumulative union rates of 86% upon literature review, with most cases in the lower extremity [2]. Despite its prevalence in the lower extremities, case reports and series on the successful use of the Masquelet technique are also described in the upper extremity. These studies have described its use in treating bone loss secondary to trauma, osteomyelitis, or nonunion, as well as for arthrodesis or septic arthritis [3-9]. In this article, we present the case of a patient who sustained a gunshot wound resulting in traumatic bone loss, articular surface destruction, and subsequent osteomyelitis following initial treatment, and ultimately underwent the Masquelet procedure with successful arthrodesis of the distal interphalangeal (DIP) joint as an alternative to digit amputation. We informed the patient that the data concerning the case would be submitted for publication and he agreed.

## Case presentation

**Figure 1:**
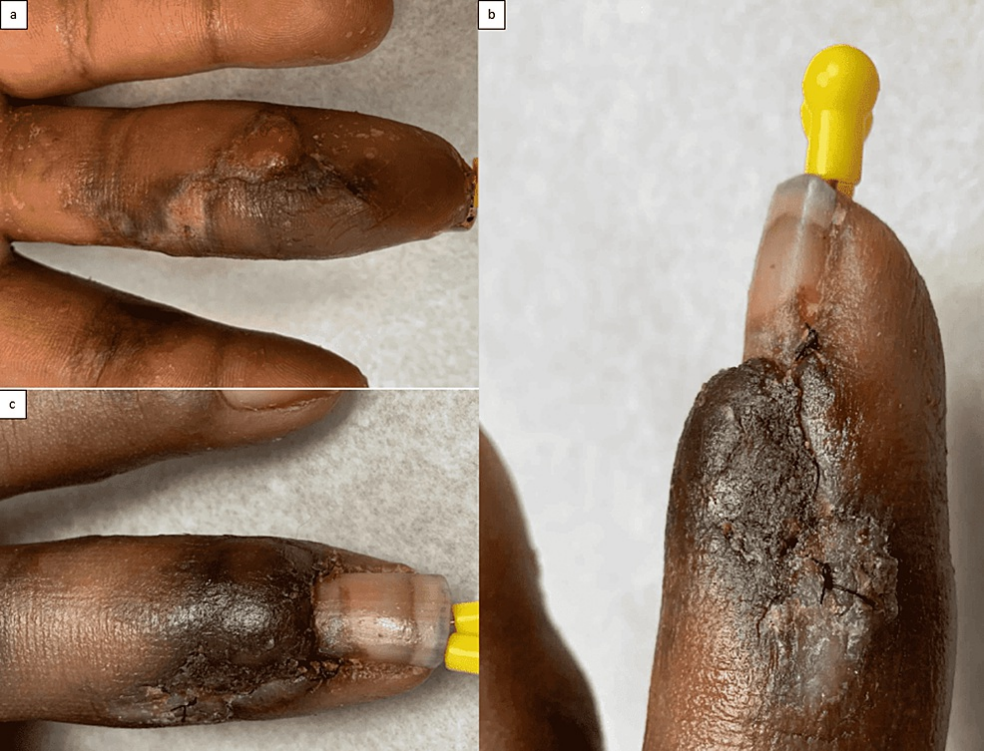
Preoperative images of the infected digit. Palmar (a), oblique (b), and dorsal views (c).

**Figure 2:**
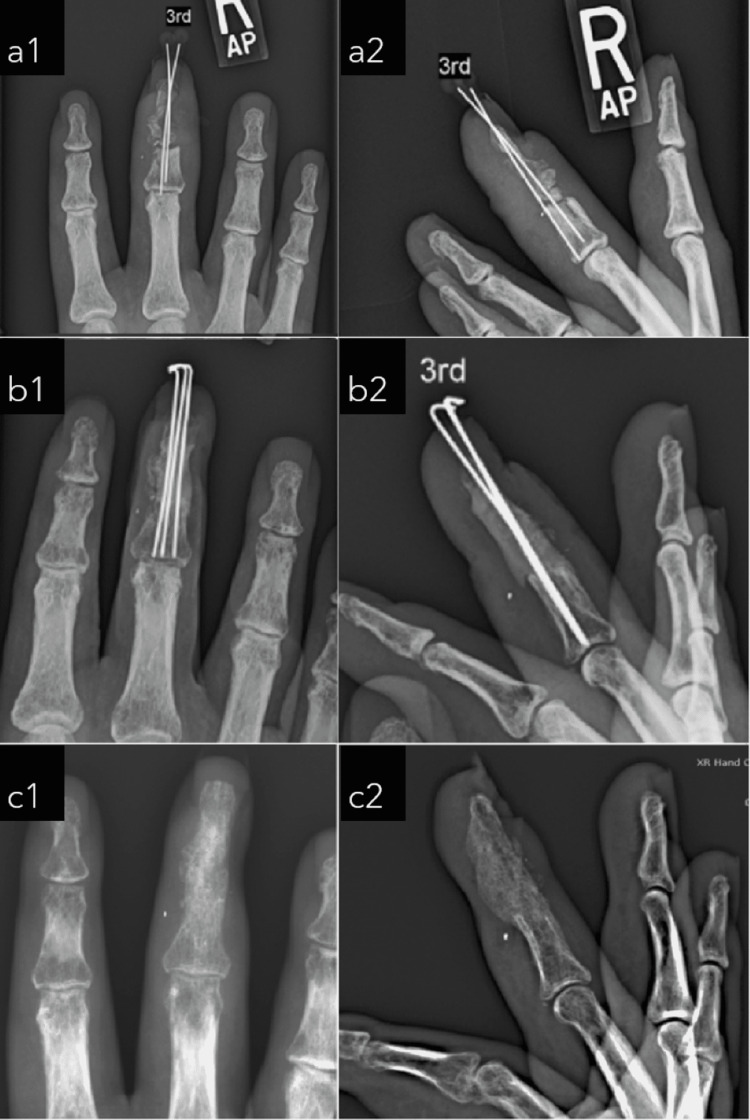
Posterior-anterior and lateral radiographs at preoperative (a1, a2), post first stage (b1, b2), and post second stage (c1, c2).

A 36-year-old left-hand-dominant man presented with a gunshot wound injury to his right long finger that he suffered two months prior. Three weeks after his injury, the patient underwent initial surgery at an outside institution consisting of irrigation and debridement of the wound as well as pinning to maintain alignment and length. Past medical history was notable for high blood pressure, high cholesterol, obesity, and obstructive sleep apnea. Physical examination of the right long finger revealed swelling from the midportion of the proximal phalanx extending to the distal phalanx. He had a well-healed ulnar-sided incision along the course of the digit extending from the distal portion of the proximal phalanx to the dorsum of his DIP joint. The capillary refill was intact, and the finger was warm. There was mild granulation through a small draining wound along the palmar aspect of the middle phalanx. Active range of motion (ROM) demonstrated minimal mobility of the proximal interphalangeal (PIP), metacarpophalangeal (MP), and DIP joints. The digital sensation was subjectively intact as he noted that his sensation was “normal” through the entirety of the digit. There was rotational deformity of the digit with pronation and shortening of the distal phalanx. Radiographs demonstrated comminuted and displaced fractures of the distal half of the middle phalanx with no viable DIP joint remaining. Two retrograde K-wires extended from the distal phalanx to the base of the middle phalanx (Figures 1, 2).

We recommended digit amputation to treat his injury, but he adamantly refused. Alternatively, we proposed a two-stage procedure with the Masquelet technique in order to salvage the digit and informed him that this treatment would not guarantee a functional digit. He expressed understanding of the expected outcomes and elected to proceed with the alternative recommendation.

**Figure 3:**
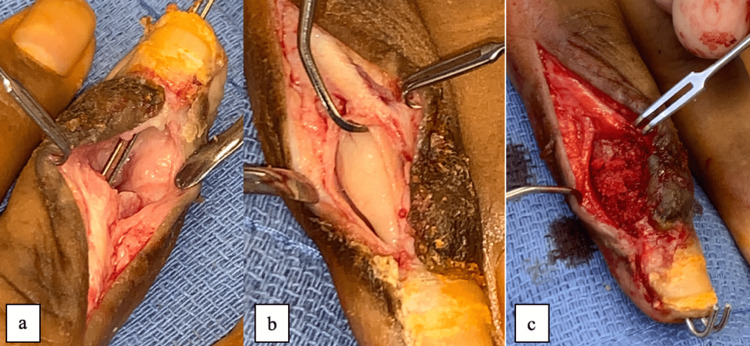
Intraoperative images of the (a) debridement (first stage), (b) placement of antibiotic cement spacer (first stage), and (c) placement of the iliac crest bone autograft.

Operative treatment at our institution was performed in two stages by a fellowship-trained orthopedic hand surgeon. The first stage, completed under tourniquet, included thorough soft tissue debridement, removal of devitalized bony fragments, insertion of an antibiotic spacer, removal of hardware, and internal fixation via three retrograde 0.9 mm Kirschner (K) wires (Figure 3). Intraoperative cultures were positive for methicillin-resistant *Staphylococcus aureus*. The antibiotic spacer consisted of polymethylmethacrylate mixed with vancomycin (1 g) and tobramycin (1.2 g). An Esmarch bandage was trimmed and utilized to form the cement into a tube around the K wires to prevent cement extravasation and protect surrounding tissues. He underwent the second stage of the surgery eight weeks following the initial procedure, also under tourniquet, which consisted of removal of the antibiotic spacer, placement of iliac crest bone graft, arthrodesis of the DIP joint, and adjustment of internal fixation. The three 0.9 mm K wires were replaced with two 1.1 mm K wires following bone graft insertion. We closed the surgical incision without issues with a 4-0 nylon suture with a combination of interrupted horizontal mattress and simple fashions. He received supplemental oral antibiotics for six weeks with linezolid as he refused intravenous antibiotic administration.

He offered no concerns following the second-stage procedure, and both surgical sites healed without complications. We followed the patient over the next eight months confirming radiographic bony healing and improvement in the ROM of the MP and PIP joints after therapy. He reported no concerns of pain, recovered the ability to forcefully grip with a marked improvement in MP joint mobility to 45 degrees of flexion and minimal improvement in PIP joint mobility, and was satisfied with the cosmetic appearance of the finger with the reestablishment of length and collinear alignment to the adjacent digits.

## Discussion

The treatment of mutilated hand injuries is complex with potential injuries to vascular, skeletal, musculotendinous, neurologic, and cutaneous structures. Hand surgeons must attempt to restore functionality and esthetics with a focus on a hand with intact sensation, and a pain-free, powerful grasp [10]. Moris et al. retrospectively identified 18 patients with traumatic bone loss or nonunion following trauma of the phalanx or metacarpal treated with the Masquelet procedure. Bone union was obtained in 89% of these patients, suggesting the Masquelet procedure to be an effective form of surgical treatment for the distal upper extremity in cases of segmental bone loss from trauma [5]. Aimé et al. reported a case series of 13 digits treated with single-stage antibiotic cement spacers for osteomyelitis with joint involvement and requiring segmental bone excision for digit salvage. Ten of these digits were successfully treated by the procedure, and all 13 of the digits were successfully salvaged [9]. Our case represents a combination of these two techniques for a combination of both indications, and to these authors’ knowledge, this has not been reported in the literature. Further, our case utilized the Masquelet technique to achieve arthrodesis of the DIP joint. There is limited research describing the use of the Masquelet technique to achieve arthrodesis in the hand following osteomyelitis, and none of the previously published reports included patients with traumatic segmental bone loss as was present in our report [7,8]. Our technique required the fusion to be performed in full extension. Villani et al. report fusion in full extension typically leads to acceptable cosmetic outcomes but can hinder flexion of the other digits [11]. Despite this, patients typically adapt to maximize their function [12]. A systematic review on DIP arthrodesis in 2014 concluded that the literature describes a wide variety of optimal angles of fusion without enough high-quality evidence to support a specific technique or angle of fusion [13].

Toyama et al. utilized the induced membrane technique for the treatment of osteomyelitis of the phalanges in seven patients. In this report, a four-week interval was used between cement spacing and bone graft for early rehabilitation and decreased burden on the patient [3]. However, Tabib et al. reported on the treatment of chronic osteomyelitis of the second metacarpal following treatment of a Bennett fracture via the induced membrane technique with an eight-week interval between stages and successful bony union [6]. Compared to our case, the previous two reports were not for traumatic segmental bone loss and did not utilize a temporary antibiotic cement spacer as described in this report. Further, our patient was unique in that he was previously recommended for amputation given the extent of bone loss, joint destruction, loss of function, and underlying infection. He adamantly refused an amputation and insisted on salvage of the digit, which led to alternative treatment via surgical debridement, placement of an antibiotic cement spacer, subsequent removal of the antibiotic cement spacer, then iliac crest bone grafting and arthrodesis of the DIP joint eight weeks after the primary salvage procedure.

Despite the success of the Masquelet technique in digit salvage, the question remains if the patient received the optimal care by declining amputation. Several reports have previously described the expected outcomes of hand function following digit amputation [14-18]. All these studies report significant loss of grip and pinch strength. Chow et al. report sensory disturbances at one year of cold intolerance (19.4%), numbness (50.1%), and hyperesthesia (13.9%). They also report a loss of ROM at the joint proximal to the amputation of 15% compared to the contralateral side [14]. Peimer et al. provided ray amputation patients with a questionnaire in their study which demonstrated that most patients are subjectively satisfied after amputation with regard to appearance, function, sensibility, and return to work after surgery [15]. Our patient had a loss of full ROM at the final follow-up but reported no sensory disturbances and was subjectively satisfied with the cosmetic and functional outcome of his hand.

Limitations to our study include a lack of objective measures of hand function and a lack of follow-up beyond eight months. The patient was incarcerated during his treatment, complicating his long-term follow-up. However, maximum medical improvement was declared at his final evaluation.

## Conclusions

The Masquelet procedure with arthrodesis represents an effective method of treating segmental bone loss in the setting of osteomyelitis and joint destruction as an alternative to digit amputation. We present a case report of successful treatment for this unique indication but recommend further research into broader indications for the Masquelet technique. In conclusion, we find the Masquelet technique to be a versatile procedure for digit reconstruction.
